# Predicting poor peripheral blood stem cell collection in patients with multiple myeloma receiving pre-transplant induction therapy with novel agents and mobilized with cyclophosphamide plus granulocyte-colony stimulating factor: results from a Gruppo Italiano Malattie EMatologiche dell’Adulto Multiple Myeloma Working Party study

**DOI:** 10.1186/s13287-015-0033-1

**Published:** 2015-04-17

**Authors:** Pellegrino Musto, Vittorio Simeon, Alberto Grossi, Francesca Gay, Sara Bringhen, Alessandra Larocca, Roberto Guariglia, Giuseppe Pietrantuono, Oreste Villani, Giovanni D’Arena, Carmela Cuomo, Clelia Musto, Fortunato Morabito, Maria Teresa Petrucci, Massimo Offidani, Elena Zamagni, Paola Tacchetti, Concetta Conticello, Giuseppe Milone, Antonio Palumbo, Michele Cavo, Mario Boccadoro

**Affiliations:** Scientific Direction, IRCCS, Referral Cancer Centre of Basilicata, Rionero in Vulture, PZ Italy; Laboratory of Pre-clinical and Translational Research, IRCCS, Referral Cancer Centre of Basilicata, Rionero in Vulture, PZ Italy; Haematology, Centro Oncologico Fiorentino, Florence, Italy; Myeloma Unit, AOU Città della Salute e della Scienza, Turin, Italy; Haematology and Stem Cell Transplantation Unit, IRCCS, Referral Cancer Centre of Basilicata, Rionero in Vulture, PZ Italy; Transfusional Medicine, IRCCS, Referral Cancer Centre of Basilicata, Rionero in Vulture, PZ Italy; Transfusional Service, S. Carlo Hospital, Potenza, Italy; Haematology Unit, AO Cosenza, Cosenza, Italy; Haematology Unit, La Sapienza University, Rome, Italy; Clinica di Ematologia, AOU Ospedali Riuniti, Ancona, Italy; Seràgnoli Institute of Haematology, University School of Medicine, Bologna, Italy; Department of Clinical and Molecular Biomedicine, Section of Haematology, University of Catania, Catania, CT Italy; Hemopoietic Transplant Program, AOU Policlinico Vittorio Emanuele, Catania, Italy

## Abstract

**Introduction:**

A still not well defined proportion of patients with multiple myeloma (MM) and eligible for autologous stem cell transplantation (AuSCT) fails to mobilize CD34+ peripheral blood stem cells (PBSC) at all or to collect an adequate number for a safe procedure or sufficient for multiple transplants. These so-called “poor-mobilizers” are difficult to be predicted, due to marked difference across previous heterogeneous studies.

**Methods:**

We aimed to develop a method based on simple clinical parameters for predicting unsuccessful (<2 × 10^6^/kg) or sub-optimal (<5 × 10^6^/kg) collections of CD34+ PBSC in newly diagnosed MM patients eligible for AuSCT, treated with novel agents and receiving an homogeneous mobilizing therapy with cyclophosphamide and granulocyte-colony stimulating factor (G-CSF). To this purpose, 1,348 patients enrolled in five consecutive Italian clinical trials were retrospectively analysed. Age, baseline low peripheral blood cell counts, use of lenalidomide, and haematological toxicity developed during induction were taken into account as possible factors associated with poor mobilization.

**Results:**

Overall, 280 patients (20.8%) showed either sub-optimal (167 patients, 12.4%) or unsuccessful (113 patients, 8.4%) collections. All analysed parameters negatively influenced the procedure, but only age and haematological toxicity during induction maintained their significance at multivariate analysis. Based on ordinal logistic regression model, we constructed a risk heat-map where the four parameters were pooled and weighted according to their relevance as single or combined variables. This model was predictive for different probabilities of failure, suboptimal or optimal outcomes.

**Conclusions:**

We found that about one fifth of newly diagnosed MM fails to collect an adequate number of PBSC. Our model, based on a large group of patients treated frontline with novel agents and receiving the most popular mobilizing approach currently employed in Europe, is applicable in individual subjects and may contribute to the early identification of “poor mobilizer” phenotypes.

**Electronic supplementary material:**

The online version of this article (doi:10.1186/s13287-015-0033-1) contains supplementary material, which is available to authorized users.

## Introduction

Multiple myeloma (MM) is a neoplastic plasma cells disorder accounting for approximately 13% of all hematologic malignancies [1]. In Europe, melphalan-prednisone (MP) plus thalidomide (MPT) or bortezomib (MPV) represent the initial standards of care for elderly MM patients [2,3], while induction therapy including novel agents followed by autologous stem cell transplantation (AuSCT) is the gold standard for frontline treatment of younger, transplant-eligible subjects [3,4]. Notwithstanding, a variable proportion of these last patients fails to mobilize CD34+ peripheral blood stem cells (PBSC) at all, or to collect an adequate number of these cells for a safe AuSCT or sufficient for additional transplants [5-7]. The percentage of these ‘poor mobilizers’, however, differs across studies, depending on definitions, parameters utilized to evaluate collections, age, disease type, phase and characteristics, treatments applied, objectives to reach, and practices for mobilization and apheresis [8,9]. Due to such heterogeneity, data are difficult to analyze and to compare.

This study aimed to evaluate the rate of unsatisfactory CD34+ PBSC collections and to investigate the possible role of some easily available clinical parameters in predicting this phenomenon in MM patients eligible for AuSCT, treated at diagnosis with novel agents and homogeneously mobilized with cyclophosphamide and granulocyte-colony stimulating factor (G-CSF), which is the principal approach currently employed in Europe.

## Methods

Overall, 1,348 newly diagnosed patients with MM enrolled in five consecutive clinical trials conducted by GIMEMA/Multiple Myeloma Italian Network were retrospectively evaluated [10-14]. According to the different study protocols, induction regimens consisted of: 1) thalidomide + dexamethasone (TD: 316 patients; clinical trial number NTC01341262 – clinicaltrials.gov, registered 11 January 2004); 2) bortezomib, thalidomide and dexamethasone (VTD: 258 patients; clinical trial number NCT01134484 – clinicaltrials.gov, registered 28 May 2010); 3) lenalidomide and dexamethasone (RD, 396 patients; clinical trial numbr NCT00551928 – clinicaltrials.gov, registered 30 October 2007); 4) pegylated liposomal doxorubicin, bortezomib, and dexamethasone (PAD, 86 patients; clinical trial Eudract number 2005-004714-32 – AIFA, registered 30 September 2005); 5) a further group of 292 patients receiving a modified VAD induction regimen (doxorubicin, vincristine and dexamethasone), not including novel agents (clinical trial number AOGIBAT-III-2004-002, registered 07 May 2004) was also evaluated. In all these patients the impact on CD34+ PBSC collection of: 1) age (>60 years), 2) initial use of lenalidomide (up to four cycles), 3) cytopenia at diagnosis (Hb <10 g/dl, neutrophil count <1 × 10^9^/L, platelet count <100 × 10^9^/L: at least one), and 4) grade 3 to 4 hematological toxicity during induction therapy (CTCAE v4) was analyzed. All these parameters are generally believed potentially able to negatively affect releasing of PBSC and to impair their collection in MM [15-18]. Initially, we also considered extensive radiotherapy to marrow bearing tissue, which was, however, excluded in the final analysis, as the number of patients who underwent such a treatment was very small and almost entirely comprised within the group developing cytopenia during induction.

In all cases, the mobilizing regimen was cyclophosphamide (3 to 4 g/sqm) + G-CSF (10 mcg/kg), given in two daily divided doses, without the addition of the mobilizing agent plerixafor, a drug still not available at the time of the evaluated trials. The morning dose of G-CSF was omitted on the harvest day. An absolute CD34+ PBSC number >20/μl was the threshold to start apheresis. Due to the retrospective nature of the study, which included patients enrolled over a prolonged period of time, apheresis machines, characteristics and methods of collection were progressively adjusted, according to available standards. In most patients, however, the amount of blood processed was twice the calculated total blood volume, by using Cobe Spectra or Fresenius AS series machines, and the target for CD34+ PBSC harvest was at least 5 × 10^6^/kg. CD34+ PBSC total amounts after a single mobilization procedure <2 × 10^6^/kg (the minimum target cell dose usually required to proceed to AuSCT) and >2/<5 × 10^6^/kg (a cell dose ensuring a safe and rapid marrow recovery, as well as the possibility to perform at least two AuSCT) were considered ‘failures’ or ‘sub-optimal’ results, respectively. Data were collected from two databases (Turin and Bologna). No formal ethical approval and/or consent form were needed for this study, according to current Italian law, as it was a retrospective, observational study, which was, however, performed according to Helsinki Declaration principles for experimental research on humans.

All risk factors were analyzed by univariate and multivariate logistic regression, taking into consideration two models having as outcome the risk to have a ‘failure’ or that of a ‘sub-optimal’ collection. The results were internally validated using the bootstrap method. In addition, receiver operating characteristic (ROC) curves were constructed to assess model discriminatory power for the predictive probability. The proportional odds assumption was evaluated by the Brant test. Finally, predicted probabilities of outcomes, based on the ordinal logistic regression model (optimal versus suboptimal versus failure), were used to generate a patient-based risk heat-map where the four parameters were pooled and weighted according to their relevance as single or combined variables. Statistical analysis was performed using the STATA analysis program, version 11.0 (Stata Corp., College Station, TX, USA).

## Results

The distribution of analyzed parameters in the whole population is reported in Table 1. Among the 1,348 patients evaluated, 402 (29.8%) had no ‘negative’ parameters, while 946 (70.2%) showed at least one of them; in particular, 630 patients (46.7%) had only one parameter, while 319 patients showed a combination of two (252, 18.7%), three (54, 4.0%) or four (10, 0.7%) parameters, respectively. Overall, 560 patients (41.5%) were more than 60 years old, 332 patients (24.6%) had baseline cytopenia, 356 patients (26.4%) were treated with lenalidomide and 88 patients (6.5%) developed grade 3 or 4 hematological toxicity under induction therapy.

**Table 1:** **Distribution of the analyzed parameters in the whole population**

**Parameters**	**Total number (%)**
**Age**	
<60 years	788 (58.46%)
≥60 years	560 (41.54%)
**Lenalidomide use**	
No	992 (73.59%)
Yes	356 (26.41%)
**Hematological toxicity**	
No	1260 (93.47%)
Yes	88 (6.53%)
**Baseline cytopenia**	
No	1016 (75.37%)
Yes	332 (24.63%)
**Number of parameters**	
0	402 (29.82%)
1	630 (46.74%)
2	252 (18.69%)
3	54 (4.01%)
4	10 (0.74%)

Distribution of the four analyzed parameters potentially affecting PBSC collection in 1,348 newly diagnosed myeloma patients. Lenalidomide use: up to four cycles; baseline cytopenia: Hb <10 g/dl, neutrophil count <1 × 10^9^/L, platelet count <100 × 10^9^/L: at least one at diagnosis; hematological toxicity: grade 3 or 4 during induction therapy (CTCAE v4). PBSC, peripheral blood stem cells.

**Figure 1:**
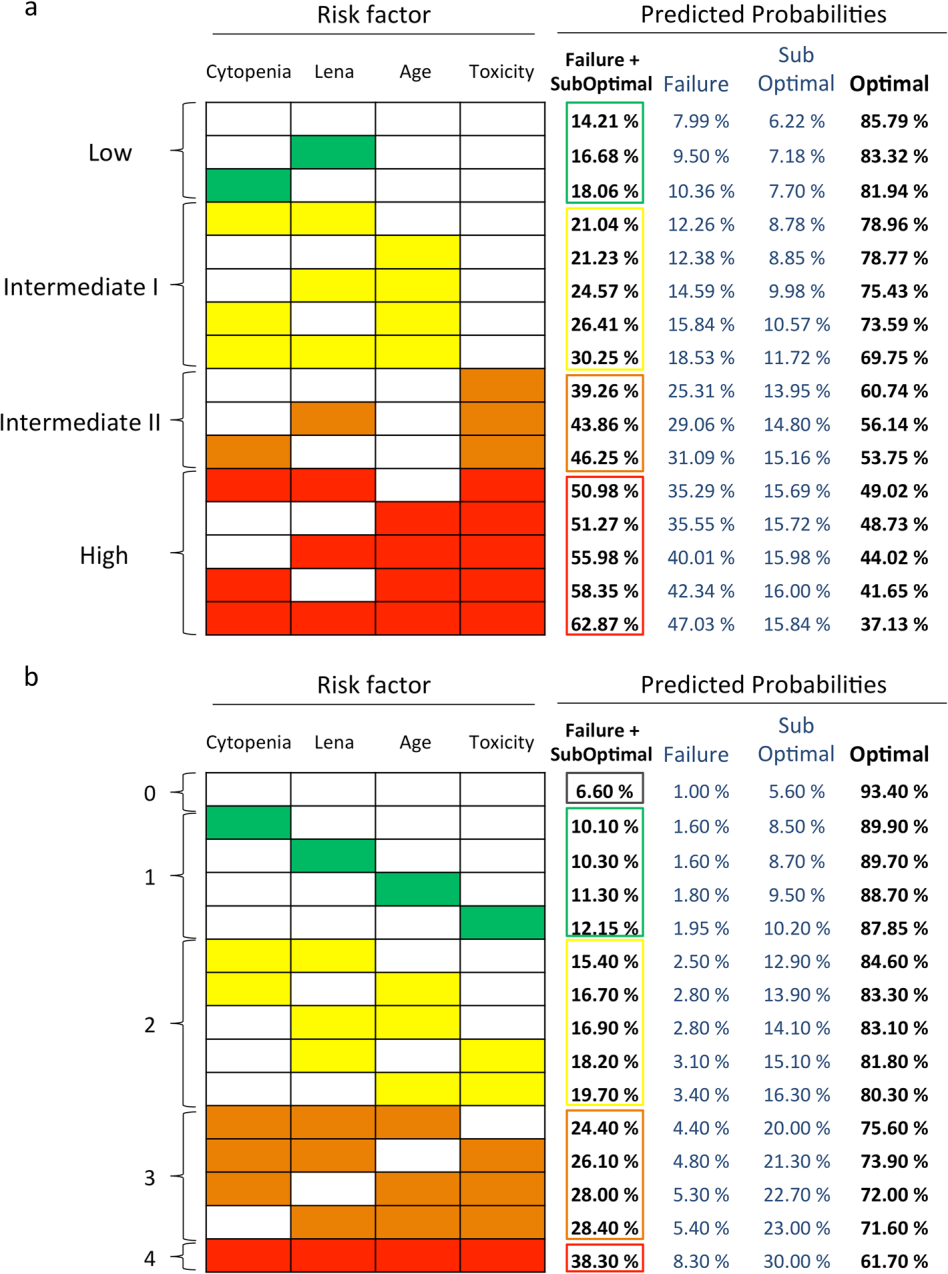
**Predictive risk heat-map. a)** Predictive risk heat-map, applicable in individual patients, in which probabilities of failures, suboptimal and optimal collections, according to relevance of risk factors as single or combined variables, were distributed in four different and growing (from green to red) risk areas. Data were generated in the whole population of 1,348 newly diagnosed multiple myeloma patients; **b)** Predictive risk heat-map in patient population (number, 1,203, 89.24%) with an absolute number of circulating CD34+ PBSC >20/μl at the time of apheresis. PBSC, peripheral blood stem cells.

After a single mobilizing procedure (median of leukapheresis: 2; range: 0 to 4), 280 patients (20.8%) collected an insufficient number of CD34+ PBSC, including 167 patients (12.4%) with unsuccessful and 113 patients (8.4%) with sub-optimal collections, respectively (Table 2). An ordinal logistic regression model showed that each single parameter negatively influenced collections at univariate analysis (Table 3); at multivariate analysis, however, only hematological toxicity developed during induction and age ≥60 years maintained a statistically significant negative effect on mobilization (Table 3). Data regarding the effect of other induction regimens on PBSC collection indicated that the use of agents such as bortezomib, thalidomide and doxorubicin did not negatively influence PBSC collections (data not shown). Figure 1a illustrates the risk heat-map obtained based on an ordinal logistic regression model. According to the cumulative probability of unsuccessful collections (failures + suboptimal), four different areas were identified, respectively, at low (range 14% to 18%), intermediate-1 (21% to 30%), intermediate-2 (39% to 46%), and high (50% to 63%) risk. In particular, the possibility of a complete failure ranged from 9% for lowest-risk, to 40% for highest-risk patients. Interestingly, the number of parameters also paralleled the risk of unsatisfactory collections in 1,203 patients (89.24% of the entire population) reaching 20/μl (or more) circulating CD34+ PBSC at the time of apheresis (Figure 1b). All these findings did not differ significantly when the 294 patients who had not received novel agents were excluded from the analysis (data not shown). Finally, ROC curves, based on the logistic regression model (Additional file 1), depicted an area under the curve (AUC) of 0.63 and 0.62, respectively, in discriminating patients with failure or sub-optimal outcome (Additional file 2).

**Table 2:** **Distribution of failure, suboptimal and optimal collections in the whole population**

**Parameters**	**Failure**	**Sub-Optimal**	**Optimal**
	**Number (%)**	**Number (%)**	**Number (%)**
**Whole population**			
Outcome	167 (12.39)	113 (8.38)	1068 (79.23)
**Age**			
<60 years	80 (10.15)	57 (7.23)	651 (82.61)
≥60 years	87 (15.54)	56 (10.00)	417 (74.46)
**Lenalidomide use**			
No	113 (11.39)	78 (7.86)	801 (80.75)
Yes	54 (15.17)	35 (9.83)	267 (75.00)
**Hematological toxicity**			
No	132 (10.48)	106 (8.41)	1022 (81.11)
Yes	35 (39.77)	7 (7.95)	46 (52.27)
**Baseline cytopenia**			
No	117 (11.52)	80 (7.87)	819 (80.61)
Yes	50 (15.06)	33 (9.94)	249 (75.00)

Absolute numbers and percentages of failures, suboptimal and optimal collections, according to the presence of different risk factors in 1,348 newly diagnosed myeloma patients. Failure: CD34+ PBSC <2 × 10^6^/kg; Suboptimal: CD34+ PBSC >2 and <5 × 10^6^/kg; Optimal: CD34+ PBSC >5 × 10^6^/kg. PBSC, peripheral blood stem cells.

**Table 3:** **Ordinal logistic regression model**

**Parameters**	**Univariate analysis**			**Multivariate analysis**		
	**OR**	**95% CI**	***P***	**OR**	**95% CI**	***P***
Age	1.63	1.25 to 2.12	0.0001	1.62	1.24 to 2.13	0.0001
Lenalidomide use	1.4	1.05 to 1.85	0.021	1.2	.89 to 1.63	0.217
Hematological toxicity	4.51	2.92 to 6.97	0.0001	3.9	2.48 to 6.14	0.0001
Baseline cytopenia	1.38	1.03 to 1.85	0.029	1.33	.98 to 1.8	0.062

Univariate and multivariate analysis of risk factors analyzed. Statistical significance was defined as *P* <0.05. CI, confidence interval; OR odds ratio.

## Discussion

An insufficient availability of PBSC may be a relevant clinical problem in patients otherwise eligible for AuSCT, a procedure that represents the best therapeutic approach in MM. We quantified this phenomenon, showing that a significant proportion of previously untreated MM patients (at least one out of five) completely fails CD34+ PBSC collection or does not reach an adequate number after a standard mobilizing procedure. Our results also show that older age, baseline cytopenia and severe hematological toxicity during the induction treatment (which likely suggest a more compromised marrow function) may be useful to highlight patients at risk of an impaired release of PBSC in newly diagnosed MM. Of interest, in our series, some patients with hematological toxicities due to induction treatment also showed one or more cytopenias at the time of diagnosis, before any treatment; both of these parameters had singly a negative impact on harvest at univariate analysis. However, when the results were investigated in multivariable analysis using two different assays (ordinal outcome and logistic regression), only hematopoietic toxicity remained statistically significant, while low baseline peripheral blood cell counts did not. The use of lenalidomide has also been considered a possible risk factor, although patients receiving lenalidomide-based regimens can be mobilized with appropriate strategies [19]. In particular, a short course of lenalidomide did not affect PBSC harvest in patients treated with cyclophosphamide plus G-CSF as mobilizing therapy [20]. In that study, however, the percentage of inadequate yield (<4 × 10^6^ CD34+ PBSC) after a single mobilization was similar to that (21%) observed in our study. Overall, we confirm a possible negative effect of lenalidomide on CD34+ PBSC collection and this should be taken into consideration when planning mobilization in patients with other potential risk factors, such as cytopenia.

Predicting mobilization failure using clinical variables has been demonstrated to be often inaccurate [8]. In this setting, algorithms based on the so-called ‘pre-emptive’ or ‘just in time’ evaluation of circulating CD34+ PBSC have been shown to be useful in predicting failure, allowing an immediate and appropriate addition of plerixafor to the original mobilization regimen and resulting in improvement of PBSC collection rates [21-25]. However, even among subjects with an apparently adequate number of circulating CD34+ PBSC at the time of apheresis (>20/μl), there may be a not negligible percentage of patients (5% to 10% in the literature, about 12% in our series) still achieving poor or sub-optimal results [26,27]. As a consequence, the rate of harvest failure in MM is usually higher than the rate of mobilization failure. The majority of these discrepancies can probably be ascribed to variability in mobilization, collection efficiency, premature termination of apheresis dictated by clinical problems arising during the procedure, reduced flow from central venous catheter, or poor intra-apheresis mobilization [28]. In any case, our ‘composed’ risk model was able to identify these patients at risk of a poor harvest, although potentially considered good-mobilizers, thus selecting a further population in which the use of plerixafor could be considered.

A possible definition of ‘predicted/proven poor mobilizers’ has been recently proposed by GITMO, which employed an analytic hierarchy process able to analyze quantitative and qualitative aspects of a decision need when a poor information base is available [7,29]. This proposal, however, based on clinical and laboratory criteria, included lymphomas, as well as patients with advanced/refractory disease, multiple lines of prior treatments, failure of previous mobilization attempts, extensive radiotherapy and reduced marrow cellularity or large neoplastic involvement at the time of mobilization. Indeed, our study, in which all patients had newly diagnosed MM achieving at least a partial response after induction treatment, represents a completely different scenario.

Based on four simple clinical parameters, our model was, not unexpectedly, not completely predictive (see AUC reported in Additional file 2). Taking into consideration other variables (that is, the pharmacogenetic background of mobilizing and induction regimens) could certainly improve its performance, probably at the expense, however, of a wide application in daily practice.

## Conclusions

To conclude, about 20% of newly diagnosed MM fail to collect an adequate number of PBSC. Our simple ‘risk card’, based on the largest group of patients treated frontline with novel agents and receiving the most popular mobilizing approach currently employed in Europe so far reported in this setting, is applicable in individual patients with MM and may contribute to the early identification of ‘poor mobilizer’ phenotypes. Although certainly ameliorable, this model increases our awareness in this field, representing a novel possible framework to select patients in whom to plan alternative mobilizing strategies [30,31]. Therefore, its validation in prospective series is required, possibly even in the setting of potentially more effective chemotherapy-free-mobilizing regimens, which have been recently reported as superior to the cyclophosphamide and G-CSF combination [27,32].

## Additional files

Additional file 1:
**Logistic regression model. Univariate and multivariate analysis of risk factors, taking into consideration two models having as outcome the risk to have a ‘failure’ or that of a ‘sub-optimal’ collection.** Statistical significance was defined as *P* <0.05. Additional file 2:
**Receiver operating characteristic curves (ROC) of logistic regression model.** ROC curves assessing the model discriminatory power for the predictive probability of failure and suboptimal harvests, respectively. (PDF 160 kb)

## Abbreviations

AUC: area under the curve
AuSCT: autologous stem cell transplantation
G-CSF: granulocyte-colony stimulating factor
GITMO: Gruppo Italiano Trapianto Midollo Osseo
MM: multiple myeloma
MP: melphalan + prednisone
MPT: melphalan-prednisone + thalidomide
MPV: melphalan + prednisone + bortezomib
PAD: pegylated liposomal doxorubicin + bortezomib + dexamethasone
PBSC: peripheral blood stem cells
RD: lenalidomide + dexamethasone
ROC: receiver operating characteristic
TD: thalidomide + dexamethasone
VTD: bortezomib + thalidomide + dexamethasone
