# Supplementary material for: Predicting poor peripheral blood stem cell collection in patients with multiple myeloma receiving pre-transplant induction therapy with novel agents and mobilized with cyclophosphamide plus granulocyte-colony stimulating factor: results from a Gruppo Italiano Malattie EMatologiche dell’Adulto Multiple Myeloma Working Party study
Source: Stem Cell Res Ther. 2015 Apr 17;6(1):64. doi: 10.1186/s13287-015-0033-1 (PMC4425876; doi:10.1186/s13287-015-0033-1)
Supplement: Additional file 1: — Logistic regression model. Univariate and multivariate analysis of risk factors, taking into consideration two models having as outcome the risk to have a ‘failure’ or that of a ‘sub-optimal’ collection. Statistical significance was defined as P <0.05. [file 13287_2015_33_MOESM1_ESM.pdf]

	Failure					
	Univariate analysis			*Multivariate analysis*		
	**OR**	**95% CI**	**p**	**OR**	**95% CI**	**p**
Age	1.63	1.17 - 2.25	0.003	1.57	1.12 - 2.19	**0.009**
Lenalidomide use	1.39	.98 - 1.97	0.064	1.1	.75 - 1.6	0.6
Hematological Toxicity	5.64	3.55 - 8.97	0.001	5.07	3.11 - 8.26	**0.001**
Baseline Cytopenia	1.36	.95 - 1.94	0.09	1.22	.84 - 1.78	0.284

	Sub-Optimal					
	Univariate analysis			*Multivariate analysis*		
	**OR**	**95% CI**	**p**	**OR**	**95% CI**	**p**
Age	1.63	1.25 - 2.12	0.001	1.61	1.23 - 2.12	**0.001**
Lenalidomide use	1.4	1.05 - 1.86	0.022	1.23	.91 - 1.66	0.179
Hematological Toxicity	3.92	2.52 - 6.1	0.001	3.35	2.12 - 5.3	**0.001**
Baseline Cytopenia	1.38	1.03 - 1.86	0.029	1.33	.98 - 1.8	0.065

Additional file 1
