# Supplementary figures and images for: Predicting poor peripheral blood stem cell collection in patients with multiple myeloma receiving pre-transplant induction therapy with novel agents and mobilized with cyclophosphamide plus granulocyte-colony stimulating factor: results from a Gruppo Italiano Malattie EMatologiche dell’Adulto Multiple Myeloma Working Party study
Source: Stem Cell Res Ther. 2015 Apr 17;6(1):64. doi: 10.1186/s13287-015-0033-1 (PMC4425876; doi:10.1186/s13287-015-0033-1)

| Obs  | ROC Area | Std. Err. | 95% CI      |
|------|----------|-----------|-------------|
| 1348 | 0.6352   | 0.025     | 0.58 - 0.68 |

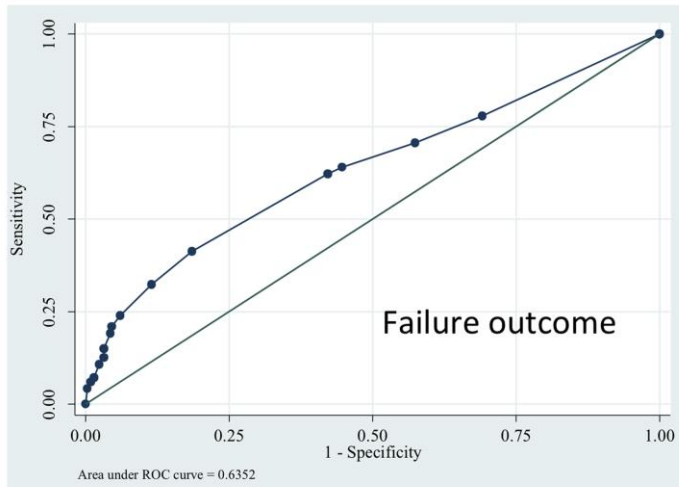

| Obs  | ROC Area | Std. Err. | 95% CI      |
|------|----------|-----------|-------------|
| 1348 | 0.6247   | 0.019     | 0.59 - 0.66 |

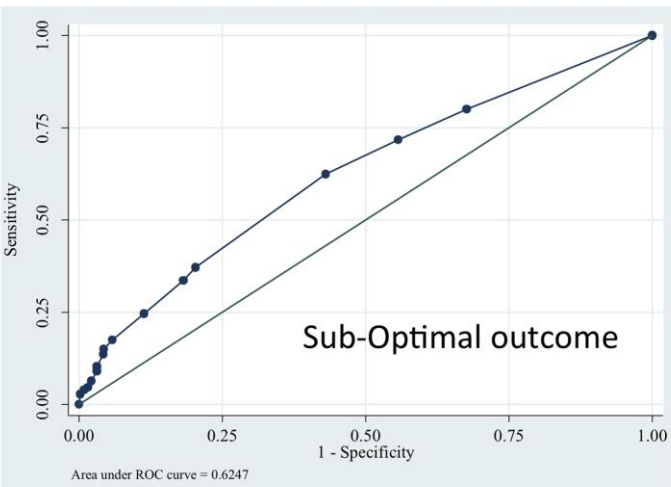

Supplement: Additional file 2: — Receiver operating characteristic curves (ROC) of logistic regression model. ROC curves assessing the model discriminatory power for the predictive probability of failure and suboptimal harvests, respectively. (PDF 160 kb) [file 13287_2015_33_MOESM2_ESM.pdf]
